# Supplementary material for: Corynebacterium accolens Has Antimicrobial Activity against Staphylococcus aureus and Methicillin-Resistant S. aureus Pathogens Isolated from the Sinonasal Niche of Chronic Rhinosinusitis Patients
Source: Pathogens. 2021 Feb 14;10(2):207. doi: 10.3390/pathogens10020207 (PMC7918835; doi:10.3390/pathogens10020207)
Supplement: Supplementary file 1 [file pathogens-10-00207-s001.pdf]

SUPPLEMENTAL TABLES AND FIGURES

Table S1. Description of oligonucleotides used for PCR amplification of *rpoB* gene in *C. accolens* isolates

Primer name	Primer sequence (5'-3')	Amplicon size (bp)
C2700F	5'-CGWATGAACATYGGBCAGGT-3'	446bp
C3130R	5'-TCCATYTCRCCRAARCGCTG-3'	

Table S2. Pathogenic *S. aureus* strains (8MSSA and 8MRSA) isolated from the sinonasal cavity of CRS patients used in this study

Strain code number	Source	Strain property
S. aureus C329	CRS nasal swab	MSSA
S. aureus C262	CRS nasal swab	
S. aureus C314	CRS nasal swab	
S. aureus C124	CRS nasal swab	
S. aureus C5	CRS nasal swab	
S. aureus C26	CRS nasal swab	
S. aureus C319	CRS nasal swab	
S. aureus C71	CRS nasal swab	
S. aureus C300	CRS nasal swab	MRSA
S. aureus C310	CRS nasal swab	
S. aureus C292	CRS nasal swab	
S. aureus C295	CRS nasal swab	
S. aureus C261	CRS nasal swab	
S. aureus C24	CRS nasal swab	
S. aureus C54	CRS nasal swab	
S. aureus C38	CRS nasal swab	
S. aureus ATCC25923	Bacterial culture collection	

Abbreviations: CRS, Chronic rhinosinusitis; MSSA, methicillin sensitive *S. aureus*; MRAS, methicillin resistant *S. aureus*

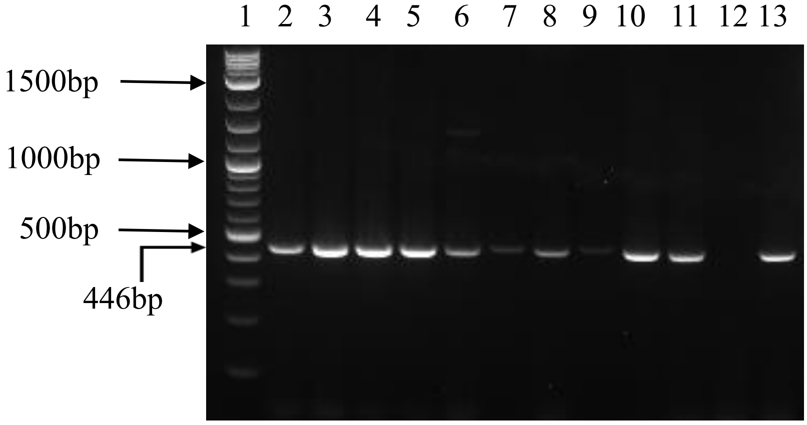

Figure S1: Identification of *Corynebacterium accolens* isolates by PCR amplification of partial *rpoB* gene (446-bp fragment). Lane 1: 1 kb plus DNA ladder. Lane 2-11: *rpoB* gene amplicon from *C. accolens* strains (C778 to C787). Lane 12: Negative control (5 µl of RNAse free water). Lane 13: Positive control (*C. accolens* ATCC49726).
